# Cultivation of Hair Matrix Cells from Cashmere Goat Skins and Exemplified Applications

**DOI:** 10.3390/ani10081400

**Published:** 2020-08-12

**Authors:** Sen Ma, Lamei Wang, Bo Zong, Ying Wang, Xiaolong Wang, Yinghua Shi, Yuxin Yang, Yulin Chen

**Affiliations:** 1 College of Animal Science and Veterinary Medicine, Henan Agricultural University, Zhengzhou 450002, China; ms0321021@163.com; 2 Henan Key Laboratory of Innovation and Utilization of Grassland Resources, Zhengzhou 450002, China; 3 Henan Engineering Research Center for Forage, Zhengzhou 450002, China; 4 Key Laboratory of Animal Genetics, Breeding and Reproduction of Shaanxi Province, College of Animal Science and Technology, Northwest A&F University, Yangling 712100, China; wanglamei1216@126.com (L.W.); zongbo1115@163.com (B.Z.); nd2013wang@163.com (Y.W.); xiaolongwang@nwafu.edu.cn (X.W.)

**Keywords:** hair follicle, hair growth, calcium, all-trans retinoic acid, HMCs, DPCs

## Abstract

**Simple Summary:**

A large scale of sequencing data pertaining to cashmere growth on cashmere goats have not been cost-effectively used due to the lack of in vitro cellular models, especially for hair matrix cells (HMCs)—the precursors of hair-forming keratinocytes, causing an enormous waste of data resources. Herein, we successfully isolated and cultivated previously unreported HMCs from cashmere goat skins and identified them morphologically and molecularly via their distinct appearance and signature genes’ expression from spatially adjacent dermal papilla cells. Through monitoring the effects of calcium and all-trans retinoic acid on HMCs using various biological techniques, we displayed that the cells are useful models to explore unsolved issues in hair fiber growth on goats. Therefore, our present success paves the road for further utilizing currently deposited data to unveil the secrets of cashmere growth and, ultimately, improve the quantity and quality of animal fibers.

**Abstract:**

A functional interpretation of filtered candidates and predicted regulatory pathways related to cashmere growth from sequencing trials needs available cell models, especially for hair matrix cells (HMCs), whose continual proliferation and differentiation result in rapid hair growth. To fulfill such goals, we herein obtained primary goat HMCs via a microdissection-based method; optimized the selection of the culture medium and coating substances for better cell maintenance; and exemplified their usefulness through examining the effects of calcium and all-trans retinoic acid (ATRA) on cells using immunoblotting, flow cytometry, and other techniques. As a result, we successfully acquired primary and passaged goat HMCs with typical keratinocyte morphology. Calcium-free RPMI (Roswell Park Memorial Institute) 1640 and MEM (minimum Eagle’s medium) outperformed normal DMEM/F12 (Dulbecco’s modified Eagle’s medium/Nutrient Mixture F-12) on long-term cell maintenance, whereas serum-free media K-SFM and EpiLife failed to support cell growth. HMCs differed molecularly and morphologically from their neighbor dermal papilla cells on expressions of feature genes, such as *HOXC13*, and on characteristic keratinocyte-like appearances versus fibroblast shapes, respectively. Higher calcium concentrations significantly stimulated the expression of the genes (e.g., *KRT1* and *IVL*) involved in keratinocyte differentiation and, promoted cell proliferation. Moreover, 10^−5^ M ATRA obviously boosted goat HMC expansions and changed their cell cycle distributions compared to the controls. Our study shines a light on researches exploring the mechanisms underlying the growth of cashmere.

## 1. Introduction

Cashmere goats are a well-recognized goat breed that is highly praised for annually yielding the precious animal fiber-cashmere from their secondary hair follicles (HFs) in skins [1]. Concurrent with HFs’ yearly sequential switching from growth stage (anagen), regression stage (catagen), and resting stage (telogen), the cashmere annually elongates and sheds [2,3]. In early efforts to delineate molecular mechanisms governing the dynamic transitions of HFs among these stages and seasonal cashmere regrowth, a large quantity of protein-coding and non-protein-coding transcripts [4,5], as well as several epigenetic modifications on genomic and post-transcriptional levels [6,7], have been filtered as potential participators via utilizing high-throughput-based sequencing strategies, such as RNA-seq. For instance, we previously performed a comprehensive transcript profiling of goat skin tissues at anagen and catagen, then simultaneously identified a total of 3500 mRNAs, 72 microRNAs (miRNAs), and 173 long noncoding RNAs (lncRNAs) that are differentially expressed between the two stages, hinting that the anagen-to-catagen transition may be under the guidance of an intricate network constituted by numerous lines of interacting coding and noncoding genes [5]. In another study, the genome-wide DNA methylomes of goat skins at anagen and telogen were examined by Li et al. using bisulfite sequencing, and they found several genes (e.g., *FMN1* and *PCOLCE*) with markedly differential methylation statuses potentially concern with the epigenetic manipulation of cashmere regeneration during the telogen-to-anagen transformation [6]. However, functional verifications of presently filtered candidates and inferred regulatory pathways generated from these studies are still relatively rare because of the shortage of suitable cell models in vitro, further hindering the deeper perception of rationales underpinning cashmere growth on goats. 

HF is a genuine complex system in anatomy, thus generally known as a mini-organ for at least the ten diverse cell types it contains [8]. Among these cells, dermal papilla cells (DPCs), hair follicle stem cells (HFSCs), and hair matrix cells (HMCs) are believed as the key components [9,10]. It is very clear that stage transitions of HF and cyclic hair growth virtually depend on dynamic alterations of these cells among finely modulated proliferation, differentiation, and apoptosis [11,12,13]. Previously, the usefulness of cultured DPCs and HFSCs serving as models to utilize presently deposited data have been demonstrated. In a recent work, Dai et al. showed that thymosin β4, a screened gene from a transcriptomic analysis, is potentially capable of enhancing cashmere production through stimulating DPCs’ proliferation [14]. Likewise, the suppressive role of miR-22-5p on HFSCs’ expansion was also revealed, exhibiting that relieving this inhibitive effect may be an alternative for goat fiber enhancement [15]. Whereas, there are still few reports focusing on the *in vitro* propagations of goat HMCs, even widespread applications (e.g., exploring the mechanisms of male baldness and detecting the dynamic expression of wool keratins) have emerged for humans [16,17] and sheep [18].

Herein, we reported the successful isolation and cultivation of follicular HMCs from cashmere goat skins. In addition, we also intentionally tested the impacts of calcium and all-trans retinoic acid (ATRA) on the proliferation and differentiation of these cells to exemplify the applicability of the obtained cells. Our success paves a solid way for efficiently taking advantage of deposited data to unravel the mystery of hair growth on goats.

## 2. Materials and Methods

### 2.1. Main Chemicals, Reagents, and Animals

Reagents and their manufacturers are listed as follows: Dulbecco’s modified Eagle’s medium/Nutrient Mixture F-12 (DMEM/F12) and EpiLife™ medium with 60-µM calcium, keratinocyte serum-free media (SFM) (K-SFM, 1×), and Coating Matrix Kit (Thermo Fisher Scientific, Waltham, MA, USA); cDNA synthesis kit, all-trans retinoic acid (ATRA), minimum Eagle’s medium (MEM) without calcium, collagen type IV, insulin, cholera toxin, epidermal growth factor (EGF), and hydrocortisone (Sigma-Aldrich, Shanghai, China); Roswell Park Memorial Institute (RPMI) 1640 medium without calcium (Genom, Hangzhou, China); Cell Cycle Analysis Kit and BeyoClick™ EdU Cell Proliferation Kit with Alexa Fluor 488 (Beyotime, Beijing, China); Chelex 100, penicillin, and streptomycin (Solarbio, Beijing, China); fetal bovine serum (FBS) (Biological Industries, Kibbutz Beit Haemek, Israel); ultraRNA pure kit (Cwbio, Beijing, China); and RealStar Green Fast Mixture (Genstar, Beijing, China). Other routine reagents were previously preserved in our labs. 

For immunoblotting, we used the following antibodies: rabbit polyclonal antibodies for ALDH1A3 (1:1000), KRT1 (1:500), SOX21 (1:250), and β-actin (1:1000) (Proteintech, Chicago, IN, USA); rabbit polyclonal antibodies for FBP1 (1:2000), OVOL1 (1:1500), KRT23 (1:200), and HOXC13 (1:500) (Sangon Biotech, Shanghai, China); and horseradish peroxidase (HRP)-labeled goat anti-rabbit IgG (H + L) (1:1000; Beyotime, Beijing, China). 

For cashmere goats, the animals were reared under the guideline of technical specifications for the feeding and management of cashmere goats (NY/T 2893-2016) issued by the Ministry of Agriculture of the People’s Republic of China. All experimental procedures were approved by the Experimental Animal Manage Committee of Northwest A&F University (Approval ID: 2013-31101684).

### 2.2. HMCs’ and DPCs’ Cultivation

**Figure 1:**
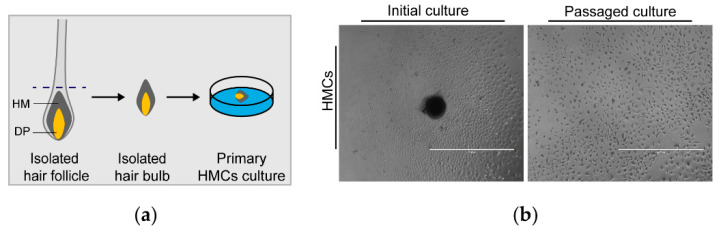
Isolation and cultivation of goat HMCs. (**a**) Schematic representation of the microdissection-based acquisition of goat HMCs from an isolated HF, and the dotted line indicates where the follicular part, including the HM and DP, was cut off using a syringe. (**b**) Primary and passaged cultures of goat HMCs. Cells outgrew from the attached explant comprising the HM and DP (black entity) and possessed a round or triangle morphology with a high nuclear/cytoplasmic ratio when passaged. HMCs, hair matrix cells; HM, hair matrix; DP, dermal papilla; and scale bar, 1000 μm.

We initially isolated the intact primary HF from female goat skin tissues incised under local anesthesia (subcutaneous injection of 1% procaine) and sterile conditions from the backside of cashmere goats. Isolation of dermal papilla (DP) explant from intact HF, in vitro propagation, and the maintenance of goat DPCs were executed as previously described [19]. To isolate the follicular matrix entity, HF were cut just above the DP using a 1-mL syringe (illustrated in Figure 1). Then, follicular matrix parts were squeezed out from entire follicular bulbs, leaving DP, dermal sheath, and out root sheath alone. Occasionally, the DP was embedded in the matrix parts. However, when DP was absent, it should be isolated again and placed very closely to the bulb matrix. Then, the bulb matrixes comprising at least one DP were carefully transferred to a 24-well culture dish coated with collagen type IV (4 μg/well) or Coating Matrix (200 μL/well) in 0.5-mL initial culture medium. Initial culture medium was basal DMEM/F12 supplemented with 10% (v/v) FBS, hydrocortisone (400 ng/mL), cholera toxin (1 nmol/L), EGF (10 ng/mL), penicillin (100 U/mL), and streptomycin (100 μg/mL). All cell cultures were kept at 37 °C in an incubator with 100% humidity and atmosphere of 5% CO_2_/95% air. After initial attachment and outgrowth, culture medium was replaced by calcium-free medium RPMI 1640 with the same supplements as the initial culture medium. At 10–14 days post-outgrowth, cells were detached from dishes using a 0.25% Trypsin-EDTA solution for 2 min and passaged to dishes precoated with collagens or others. Special care should be taken in this step, because over-digestion often causes the insufficient adhesion of passaged cells. 

### 2.3. Observation of HMCs’ Morphology and Proliferation in Different Culture Media

HMCs at the 3rd to 4th passages were seeded in triplicates (20,000 cells/well) in a 24-well culture plate, which was precoated with collagen type IV to enhance the adherent efficiency. After overnight incubation, unattached cells were removed, and the remaining cells were cultured in different kinds of medium, including DMEM/F12, MEM, and RPMI 1640 supplemented with the same additives mentioned above, and K-SFM and EpiLife, which were intentionally formulated for supporting human keratinocyte growths in vitro. At days 0, 3, and 5, the cellular morphology was digitally recorded using an inverted microscope. Then, we further counted the cell number of each culture on a hemocytometer at the 5th day to evaluate the proliferative activity of HMCs in each medium. 

### 2.4. Observation of HMCs’ Attachment and Morphology on Various Coating Substrates 

HMCs at the 3rd to 4th passages were seeded in quadruples (20,000 cells/well) in a 24-well culture plate, which were either uncoated or coated with collagen type IV (4 μg/well) or Coating Matrix protein (200 μL/well) or fibronectin (4 μg/well) or poly-L-lysine solution (200 μL/well). Cells were maintained in the incubator without disturbance for 12 h. Then, unattached cells were collected and counted on a hemocytometer to estimate the adhering efficiency. At the same time, the cellular morphology was recorded on an inverted digital microscope.

### 2.5. Growth Curve of HMCs

HMCs at the 3rd to 4th passages were seeded in triplicates in a 24-well culture plate, which was coated with collagen type IV for 12 h. When unattached cells were removed, 500-μL fresh RPMI 1640 medium was replenished for each well. Resulting cells (three wells per day) were daily harvested (from the 1st to 7th days) and counted using a hemocytometer. Meanwhile, cellular morphology was monitored as previously stated. Cell doubling time was calculated as follows: cell doubling time (h) = (duration time)/log2 (final concentration/initial concentration).

### 2.6. Treatment of HMCs with Different Concentrations of Calcium

Calcium nitrate (Ca(NO_3_)_2_·4H_2_O) powder was dissolved in sterile phosphate-buffered saline (PBS, pH = 7.4) at a concentration of 300 mM as a stock solution. Chelex 100 was strictly used as the manufacturer’s guideline to eliminate divalent calcium cations in FBS. Afterwards, RPMI 1640 medium was supplemented with the same additives as we did before, except for that chelated FBS replaced normal FBS. Different volumes of stock solution were added into RPMI 1640 medium to acquire the final calcium levels of 0.1 mM (low level), 0.5 mM (middle level), and 1.2 mM (high level). Then, these media were applied for incubating HMCs in the next two days and for performing the downstream analysis. 

### 2.7. Treatment of HMCs with ATRA

ATRA was dissolved in dimethyl sulfoxide (DMSO) with a stock concentration of 10^−2^ M and stored as the manufacturer suggested. To minimize the interfere of serum-contained ATRA and its precursors, we intentionally reduced the serum concentration to 2%, and the other additives were supplemented as usual. Then, we added 0.1% stock solution to the culture medium to achieve a final concentration of 10^−5^ M, and the same volume of DMSO was added as the control. After a two-day incubation, all HMCs were proceeded to the downstream analysis.

### 2.8. Quantitative Real-Time PCR (qRT-PCR)

Total RNAs of the cell samples were isolated using the ultraRNA pure kit according to the manufacturer’s instructions. Reverse-transcription reactions for 1-μg total RNAs were performed using the cDNA synthesis kit. QRT-PCR analysis (reaction volume: 25 μL) was performed in triplicates with the Bio-Rad IQ5 Real-Time PCR system using RealStar Green Fast Mixture according to the manufacturer’s three-step protocol. Relative levels of gene mRNA expressions were determined using the 2-∆∆Ct method, as previous studies stated [5,9]. GAPDH was set as the internal control in all experiments. Detailed information of the primers is listed in Table S1. In addition, primer specificities for each gene were judged from the melting curves of all amplicons in Table S1, in which a single peak indicates the goodness of the primers.

### 2.9. Immunoblotting

Cell lysates from culture cells (35-mm-dishes; ~200,000 cells) were prepared using Radio Immunoprecipitation Assay (RIPA) lysis buffer. Gel electrophoresis was performed using 10% sodium dodecyl sulfate-polyacrylamide gel electrophoresis (SDS-PAGE) gels and transferred to polyvinylidene fluoride (PVDF) membranes (Beyotime). Membranes were blocked in 5% (wt/vol) nonfat milk in PBS containing 0.1% Tween 20 for 2 h at room temperature (RT) and incubated with primary antibodies overnight at 4 °C and with secondary antibodies conjugated with HRP for 2 h at RT. HRP was detected using BeyoECL Plus (Beyotime, Beijing, China). Three biological replicates were set for each group. Relative expressions of proteins between or among groups were analyzed using ImageJ software. β-actin was set as the internal control to normalize the protein abundances.

### 2.10. Cell Cycle Analysis 

All cell samples were harvested and fixed in 75% ethanol. Then, cell cycles were analyzed using a Cell Cycle Analysis Kit and flow cytometry (Becton, Dickinson and Company, Franklin Lakes, NJ, USA) as their directions suggested. All data were analyzed using FlowJo software V7.6.5, and all experiments were performed in triplicates to assure good reproducibility of the results.

### 2.11. EdU Test

After each treatment, goat HMCs were incubated in fresh medium added with 10-μM EdU for an additional 4 h. Subsequent procedures were performed as the operational instruction of the BeyoClick™ EdU Cell Proliferation Kit with Alexa Fluor 488 suggested. Resulted images were recorded and analyzed using Image-Pro Plus 6.0 software. Cells stained green were considered as the proliferating cells, and all cells were stained blue (nuclear staining). Percentage of green cells in blue cells was calculated as the ratio of proliferating cells. Overall, five images randomly captured from each well were used for statistical analysis. All experiments were performed in triplicates. 

### 2.12. Data Analysis 

All data here were presented as the mean ± standard deviation (SD). Statistical analysis was performed using GraphPad Prism 6.7 software. The two-tailed *t*-test was used to compare the mean values between two groups; one-way ANOVA was applied for comparisons of the means among three groups. The criterion for statistical significance was *p* < 0.05, and statistical extreme significance was *p* < 0.01. All experiments were performed in triplicates or more to assure the reproducibility of the results.

## 3. Results

### 3.1. Outgrowth of HMCs from Isolated Hair Bulb Explants

To acquire the HMC cultures from goat HF, we isolated the intact hair bulb region (comprising the hair matrix and DP) from HF and transferred it into precoated 24-well culture dishes (overall schema: Figure 1a). After 3–5 days of incubation without disturbance, HMCs emigrated out from the edge of the attached explant (Figure 1b, left panel) and began fast proliferation. We further detached and passaged the initial cultures of HMCs following another 6–8 days of propagation to expand the cell population. As a result, HMCs in the second passage dramatically exhibited a round or triangle morphology with a high nuclear/cytoplasmic ratio (Figure 1b, right panel). These results are highly in accordance with previous reports on humans and sheep [18,20], thus strongly suggesting the feasibility of cultivating presumed HMCs from goat skins.

### 3.2. Optimization of Culture Condition for Goat HMCs

**Figure 2:**
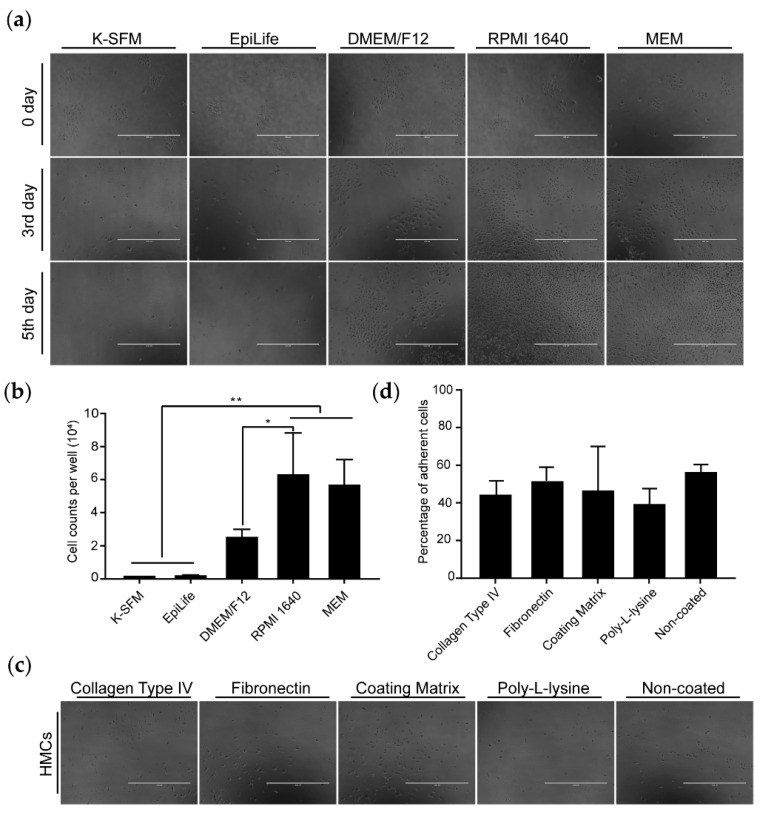
Culture condition optimization for goat HMCs. (**a**) Cells observably disappeared for EpiLife and K-SFM (serum-free media), markedly propagated in Roswell Park Memorial Institute (RPMI) 1640 and minimum Eagle’s medium (MEM), with a moderate effect in Dulbecco’s modified Eagle’s medium (DMEM)/F12. (**b**) Cell counting analysis showed that RPMI 1640 and MEM exert the optimal potential for HMC expansions. (**c**) Cells grown in the coating matrix and collagen type IV-coated petri dishes seemed to spread quicker than others, as judged by the triangle or polygonal shapes. (**d**) No statistical significance for the cell-adherent efficiency was met among the groups. **, *p* < 0.01 and *, *p* < 0.05; scale bar = 1000 μm.

Previous reports on humans and sheep revealed that HMCs exhibit differential proliferative capacity when incubated in different culture media and in plates precoated with several types of coating substances [18,20]. To establish better conditions for supporting goat HMC expansions, we examined the effects of five medium types and four kinds of adherent substances on long-term cell maintenance. Captured images in Figure 2a showed that, at the start of incubation (day 0), all cells possessed a similar morphology, which resembled passaged HMCs in Figure 1a. At day 3, cells in both serum-free media (i.e., EpiLife and K-SFM) changed their shapes into rounded, and it also seemed that these cells gradually underwent cell death, because their numbers visually decreased. Another three media supplemented with 10% FBS showed good performances in supporting HMC growths, as suggested by the remarkable expansion of the cell population and the more consistent cellular morphology. In contrast to calcium-free media (RPMI 1640 and MEM), a tiny part of HMCs in DMEM/F12 (calcium concentration > 1.0 mM) became round with enlarged cytoplasmic space, which is an indicator of keratinocyte differentiation [21]. At day 5, cells in RPMI 1640 and MEM nearly covered the overall surfaces of the culture dishes, whereas they were almost invisible in EpiLife and K-SFM. Quantities of harvested cells (Figure 2b) also implied that RPMI 1640 and MEM exerted better promotive effects on HMCs’ proliferation than K-SFM and EpiLife (*p* < 0.01), with a moderate impact of DMEM/F12. Present results demonstrate that RPMI 1640 and MEM are currently the more suitable media for the in vitro expansion of goat HMCs.

We also tested the adherent efficiency of goat HMCs on cell culture dishes coated with four coating matrices. Photos in Figure 2c hinted that HMCs incubated in the coating matrix and collagen type IV-coated culture dishes spread quicker when attached, and they exhibited a higher extent of morphological resemblances with HMCs in Figure 1b than others. Whereas, no statistical differences were detected on the ratios of the adherent cells among all groups (Figure 2d). These results instruct us that the coating matrix and collagen type IV may be preferentially selected as coating substances.

### 3.3. Growth Curve of Goat HMCs in RPMI 1640

**Figure 3:**
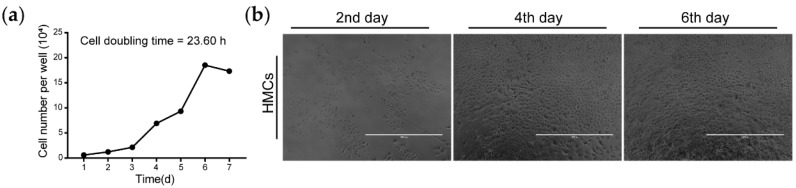
Growth curve of goat HMCs. (**a**) Cell doubling time was calculated using data at days 3 and 5 from the growth curve of goat HMCs. (**b**) Recorded images of goat HMCs at days 2, 4, and 6 displayed that partial cells are undergoing differentiation, as distinguished by the emergence of enlarged cells. Scale bar = 1000 μm.

We gained the growth curve of goat HMCs in the RPMI 1640 medium through successively cultivating them for seven days. As with other cell types, HMCs also went through a lag phase (first and second days), logarithmic phase (third–fifth days), and stationary phase (sixth day), then finally entered the decline phase (seventh day). The cell doubling time of HMCs, which is 23.60 h, was calculated through analyzing data in the logarithmic phase (Figure 3a). In addition, recorded images from different days demonstrated that part of the cells are undergoing differentiation, as shown by the appearance of enlarged cells (Figure 3b).

### 3.4. Morphological and Molecular Identifications of Goat HMCs

**Figure 4:**
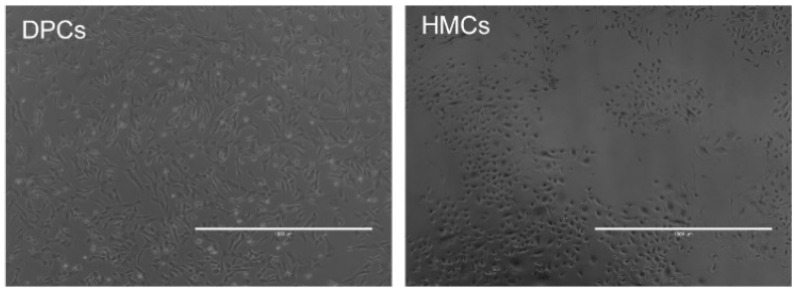
Morphological differences between DPCs and HMCs. DPCs are fibroblast-like, and HMCs are triangle or rounded. DPCs, dermal papilla cells and HMCs, hair matrix cells. Scale bar = 1000 μm.

**Figure 5:**
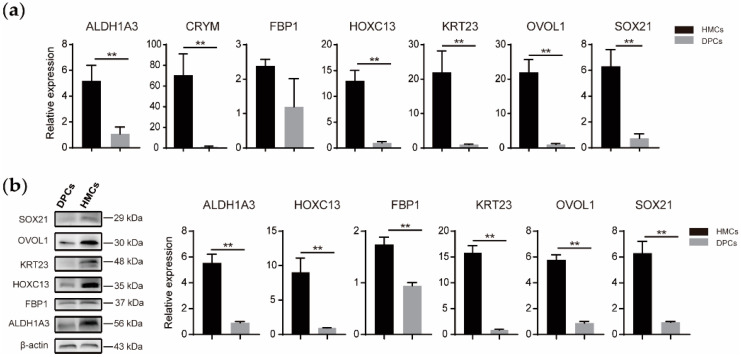
Molecular identification of goat HMCs. Expressions of several genes with crucial roles in hair growth were compared between DPCs and HMCs at the mRNA (**a**) and protein level (**b**). GAPDH and β-actin were set as the internal controls in qRT-PCR and immunoblotting experiments, respectively. Data was shown as mean ± SD. **, *p* < 0.01.

To assure the faithfulness of the obtained cell lines, we selected several functional genes (e.g., *FBP1*, *HOXC13*, and others) with indispensable characters in HF growth and compared their expression levels of mRNA and protein between HMCs and DPCs, which are positionally adjacent but virtually separated by a basement membrane within HF [8]. Firstly, these two cell types are explicitly differential in the featured exterior morphology: DPCs are fibroblast-like, whereas HMCs own a typical keratinocyte-like shape (Figure 4). Secondly, qPCR results manifested that all genes observably show higher abundances in HMCs than DPCs (Figure 5a). Among them, *ALDH1A3*, the gene encoding a key enzyme for retinoic acid synthesis [22], exhibited the highest 70-fold abundance in HMCs versus DPCs, implying that HMCs are possibly active sites of retinoid metabolism. *FBP1*, a well-recognized marker of mouse HMCs in an early study [23], also expressed as many as five times in HMCs than DPCs. A similar trend emerged in *CRYM*, a gene involved in the regulatory roles of glucocorticoids in hair growth [24]. In addition, *HOXC13*, *SOX21*, and *OVOL1* are experimentally verified as necessary transcriptional regulators of HF development, and their dysfunctions have led to observed hair loss or aberrations in mice [25,26,27]. Their abundances were higher in HMCs than DPCs, with the fold changes of 13, 21, and 22, respectively. Otherwise, *KRT23* also specifically exists in an HMC-restricted manner, with a six-fold change compared with DPCs. Similar trends were also found on the protein levels (Figure 5b).

### 3.5. Effects of Calcium on HMCs’ Proliferation and Differentiation

**Figure 6:**
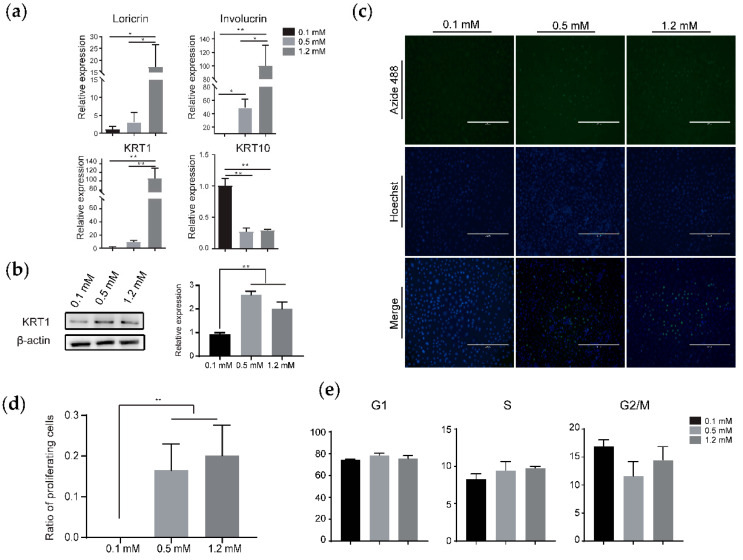
Effects of calcium on the differentiation and proliferation of goat HMCs. Calcium dramatically changed the expressions of marker genes associated with keratinocyte differentiation on the mRNA (**a**) and protein levels (**b**). Counts of proliferating cells (green) accounting for the total cells (blue) visually increased (**c**), and the ratio significantly elevated with the rising calcium concentration (**d**). Moreover, calcium altered the cell cycle distribution of goat HMCs, although it did not meet a significance criterion (**e**). Note that only the protein level of KRT1 was confirmed in (**b**). **, *p* < 0.01 and *, *p* < 0.05; scale bar = 400 μm.

The level of ionic calcium in the culture medium can markedly change the in vitro pattern of the proliferation and differentiation of keratinocytes originating from animal skin tissues [28]. To ascertain whether the concentration of ionic calcium impacts the growth of HMCs or not, we treated these cells with 0.1-mM (low level), 0.5-mM (moderate level), and 1.2-mM (high level) calcium and monitored the correspondent cellular responses using qRT-PCR, Western blotting, and other techniques. Firstly, we checked the expressions of four genes with elevated levels during the calcium-induced terminal differentiation of epidermal keratinocytes [29] and found that the abundances of three genes synchronously increased with the rising concentration of calcium in the culture media (Figure 6a). Notably, the fold changes of *loricrin*, *involucrin*, and *KRT1* ranged from at least dozens to more than a hundred in HMCs cultured in high Ca^++^ compared with low Ca^++^, verifying that calcium serves as a factor of crucial importance in the differentiation of goat HMCs. On the contrary to the three genes mentioned above, *KRT10* expressed in a decreased way when the calcium level elevated. In addition, the varied protein level of KRT1 was attested (Figure 6b), partially confirming the consistency of the expressions of the mRNA and protein. Secondly, we showed that the proliferating HMCs accounted for 16% and 22% of the total cells in the culture media with 0.5-mM and 1.2-mM calcium, respectively, but are nearly absent in low Ca^++^ (Figure 6c,d). Finally, the cell cycle examination results indicated that the proportion of HMCs at the G1 and S stages decreased in low Ca^++^ compared to the moderate or high levels, and adverse trends appeared for cells at the G2/M stage, although no statistical significances emerged in the present test (Figure 6e). Collectively, these results demonstrate that calcium is a crucial regulator in the proliferation and differentiation of goat HMCs. 

### 3.6. Stimulatory Role of ATRA on HMCs Growth

**Figure 7:**
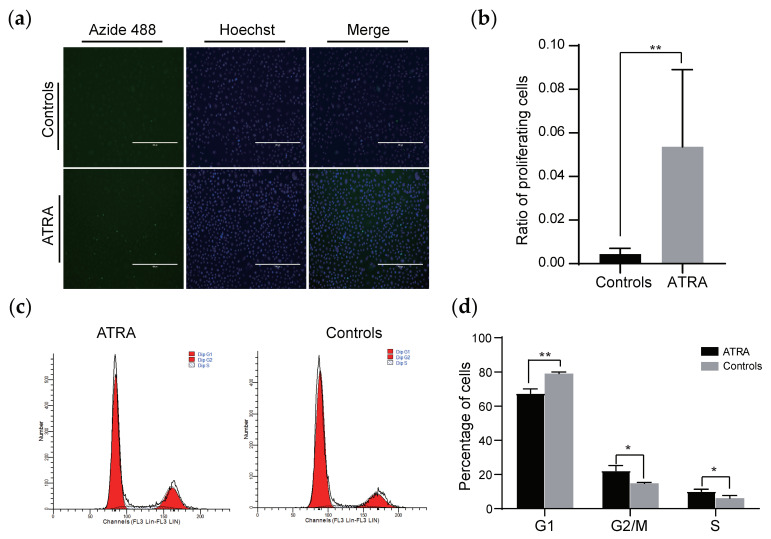
Impacts of all-trans retinoic acid (ATRA) on the proliferation and cell cycle distribution of goat HMCs. Numbers (**a**) and ratios (**b**) of proliferating cells (green) among all the cells (blue) significantly increased after treatment with ATRA (10^−5^ M). Otherwise, the cell cycle distribution of goat HMCs was drastically changed by ATRA (10^−5^ M), as shown by the quantity (**c**) and percentage (**d**) of cells located at each stage. **, *p* < 0.01 and *, *p* < 0.05; scale bar = 400 μm.

ATRA has been reportedly proposed with a promotive impact on skin keratinocyte expansions [30], whereas its roles on HMC growths are not clear yet. To investigate the potential character of ATRA on HMCs, we treated HMCs with 10^−5^ M ATRA, perceived the fluctuations in the cellular status, and analyzed the expressions of the genes involved in ATRA signaling transduction and keratinocyte differentiation. Our results pointed out that the ratio of proliferating cells upon ATRA treatment dramatically increased (Figure 7a,b), as well as the obvious changes of the cell phase distribution. ATRA significantly lowered the proportion of HMCs in G1 phase from 79.09% in the control group to 67.55% (*p* < 0.01) and propelled them into the S and G2 phases, which accounted for 14.76% and 6.15% of the total when untreated but increased to 22.32% and 10.13%, respectively, after treatment (Figure 7c,d). Then, we discovered that a 6.6- and 3.1-fold change of *CRABP1* and *RARB* expressions happened in ATRA-stimulated HMCs compared to the control (Figure S1). Moreover, the induced expression of *ivolucrin* also appeared in the ATRA-treated group (Figure S2). Taken together, these results suggested that ATRA may exert a dual role on HMCs’ growth.

## 4. Discussion

High-throughput sequencing-based strategies have greatly assisted the extensive discovery of reliable candidates involving hair growth on cashmere goats in the last decade [4,5,6,7]; however, the experimental interpretation of filtered genes extremely lags because of the scarcity of suitable cell lines. HMCs are the precursors of hair-forming keratinocytes, and their continuous proliferation and committed differentiation towards those cells result in the rapid elongation of hair during anagen (the growth phase) [31]. When the hair cycle transits from anagen to catagen (the regression phase), HMCs cease expansion, slow down differentiation, and start apoptosis, leading to the gradual termination of hair growing. Understanding the controlling mechanisms underlying these cellular activities of HMCs is key to uncover the mystery of hair growth, which urgently needs a reliable cellular model. Thus, we designed the present study to attempt to acquire goat HMCs from cashmere goat skins. We successfully gained primary cultures of these cells and established an optimized system for their passaging. In addition, we also exhibited their applicability by showing that calcium and ATRA exert differential roles on HMCs’ proliferation or differentiation, shining a bright future for cost-efficiently using generated data to clarify the enigma of cashmere growth. 

We adopted similar methods described by Bates et al. and Luo et al. to segregate hair bulbs containing hair matrix (HM) and dermal papilla (DP) from intact HFs [18,20], and goat HMCs emigrated from attached hair bulb, as expected. However, it should be mentioned that the microdissection-based separation of ideal hair bulbs needs skillful operators and time devotion, and the adhering odds of hair bulbs on culture dishes is quite low, if not so proficient, which may be the reasons why few reports focus on propagating HMCs. The blending of HM and DP cannot avoid the possibility that acquired cultures contain both DPCs and HMCs. To eliminate human DPCs, Luo et al. replaced the nutritious Chang medium with serum-free keratinocyte growth medium in 3 to 4 days post-emigration; however, this approach was not practicable for goats, as we showed that serum-free media (K-SFM and EpiLife) specifically developed for keratinocytes failed to support goat HMCs’ growth (Figure 2a). A similar phenomenon also appeared on sheep, as the growth rate of their cells drastically decreased in K-SFM [18]. Whereas, it seems that DPCs’ contamination is not observable, which could be judged from the morphology of primary and passaged cells (Figure 1b). This observation is also consistent with the result from sheep, implying that the current method is feasible for acquiring HMCs with high purity from animal skins. Further, we compared the effects of five media on promoting goat HMCs’ growth and found that calcium-free media significantly displayed better performances than DMEM/F12, which was previously utilized for propagating sheep HMCs and contains as high as 1.0 mM Ca^++^. Early reports manifest that ionic extracellular calcium induces a wide array of changes related to keratinocyte terminal differentiation, including morphology, gene expression, and growth rate [21,32], which can be ameliorated by lowering the calcium concentration for long-term keratinocyte maintenance, as K-SFM and EpiLife (0.06 mM Ca^++^) do. On the other hand, although the study hints that the addition of serum in a serum-free medium induces human HMCs’ terminal differentiation [33], it is very likely that serum deprivation-caused nutritional insufficiency is responsible for the failure of goat HMCs’ growth in a serum-free medium. This opinion is upheld by the discovery that K-SFM added with a serum achieves the desired performance on sheep HMCs’ growth [18]. Taken together, we recommend a calcium-free medium supplemented with a serum that is preferentially selected for maintaining HMCs for animals other than humans so far. 

We also found that goat HMCs are a population with high heterogeneity, as indicated by a small proportion of cells having unusual larger cell volumes and round shapes (Figure 2a and Figure 3b). This fits with the results from sheep [18] and with the truth that HMCs are mixed precursors of several follicular keratinocyte types [34]. Recently, single-cell RNA-sequencing has emerged as a powerful tool to reveal cell subtypes in complex tissues or organs [35], providing a technical solution to understand the complex nature of HMCs in later studies. 

Subsequently, comparisons of cellular morphology and gene expression between DPCs and HMCs were conducted. Both cell types own a unique appearance: DPCs are like fibroblasts, and HMCs resemble the shape of keratinocytes (Figure 4). This is in accordance with the developmental origins of both cells, that DPCs are specialized fibroblasts and HMCs arise from the skin epithelium during embryogenesis [23]. Moreover, the abundances of several HMC marker genes identified in mice show significantly higher mRNA or protein levels in goat HMCs than DPCs (Figure 5), further confirming the identity of acquired cells from the molecular aspect. At present, the functioning mechanisms of the majority of these genes with indispensable roles in hair growth are not thoroughly clarified. For instance, the essential character of *Sox21* in hair growth might link to its regulatory expressions of keratins and keratin-associated proteins [27]. Verification of these probable linkages via adopting goat HMCs as models could be a promising strategy for better interpreting the obligatory status of the genes in hair growth.

Finally, we attested the usefulness of obtained goat HMCs through examining the impacts of calcium and ATRA on cells. Low-level calcium either inhibits human HF growth in vitro [36] or causes transient alopecia with histological anomalies, including HF dystrophy and increased follicular cell apoptosis in mice [37]. Our results suggested that calcium overwhelmingly stimulates the expressions of the genes associated with keratinocyte differentiation (Figure 6a,b), indicating the modulatory roles of calcium on HMCs’ differentiation and hair formation. These agree with the specific localization of some calcium-binding proteins, such as S100A3 [38], in terminally differentiated progenies of HMCs and downregulated abundances of *K1*, *Lor*, and *Ivl* in hairless skins of mice fed a low calcium diet compared to the normal skins of mice fed a regular diet [37]. We also displayed that calcium positively affects the proliferation for goat HMCs (Figure 6c,d), which is consistent with that for sheep HMCs or immortalized human keratinocyte HaCaT [18,32]. Thus, the dual character of calcium on HMCs explains why a calcium insufficiency leads to abnormal mice phenotypes, as stated above. In addition, we demonstrated that ATRA markedly spurs goat HMC proliferation, alters their cell cycle distribution, and changes the expressions of genes involved in ATRA signaling transduction and keratinocyte differentiation (Figure 7, Figures S1 and S2). Although the above observations are a high match with the impacts of ATRA on epidermal keratinocytes [30,39], they defy the discoveries that excess accumulations of endogenous ATRA in vivo [40] or the treatment of in vitro-maintained HF with exogenous ATRA [41] causes alopecia or hair growth inhibition, respectively. Otherwise, we have experimentally elucidated that the detrimental effects are mediated by several actions of ATRA exerting on DPCs at the cellular and molecular levels [19]. Taken HF complexity into consideration, the current case indicates that the appropriate selection of cellular models is a prerequisite for correctly interpreting a defined biological phenomenon, especially when regarding less-known and conservative noncoding genes, in HF biology. Therefore, the continual enrichment and optimization of in vitro fostered follicular cell lines, as we did here, will be meaningful for meeting such a goal.

## 5. Conclusions

In present study, we successfully cultivated primary and passaged HMCs from cashmere goat skins. Calcium-free RPMI 1640 or MEM supplemented with 10% FBS and other additives are the recommended media, and the coating matrix and collagen type IV are the preferred coating substances. Goat HMCs differs substantially from DPCs either on exterior morphology or the expression of intrinsic marker genes. Inorganic calcium exerts dual roles on HMCs’ proliferation and differentiation, whereas ATRA mainly shows a stimulatory effect on HMCs’ growth. Our study provides available cellular models for cost-effectively utilizing deposited data to unravel the mystery of hair growth in goats and other animal species.

## Supplementary Materials

The following are available online at https://www.mdpi.com/2076-2615/10/8/1400/s1. Figure S1: Varied expression of ATRA signaling pathway-related genes in goat HMCs after ATRA treatment. Figure S2: Expression of marker genes for keratinocyte differentiation in goat HMCs after ATRA treatment. Table S1: Sequence information of designed primers used in the present study.

## Abbreviations

FBP1	fructose-bisphosphatase 1
ALDH1A3	aldehyde dehydrogenase 1 family member A3
CRYM	crystallin mu
HOXC13	homeobox C13
OVOL1	OVO homolog-like-1
SOX21	SRY-box transcription factor 21
KRT23	keratin 23
KRT10	keratin 10
KRT1	keratin 1
LOR	loricrin
IVL	involucrin
CRABP1/2	Cellular retinoic acid-binding protein 1/2
FABP5	fatty acid-binding protein 5
RARA/B/G	retinoic acid receptor alpha/beta/gamma
RXRA/B/G	retinoid X receptor alpha/beta/gamma
PPARD	peroxisome proliferator activated receptor delta
FMN1	formin-1
PCOLCE	procollagen C-endopeptidase enhancer 2
GAPDH	glyceraldehyde-3-phosphate dehydrogenase
