# Supplementary figures and images for: Cultivation of Hair Matrix Cells from Cashmere Goat Skins and Exemplified Applications
Source: Animals (Basel). 2020 Aug 12;10(8):1400. doi: 10.3390/ani10081400 (PMC7460477; doi:10.3390/ani10081400)

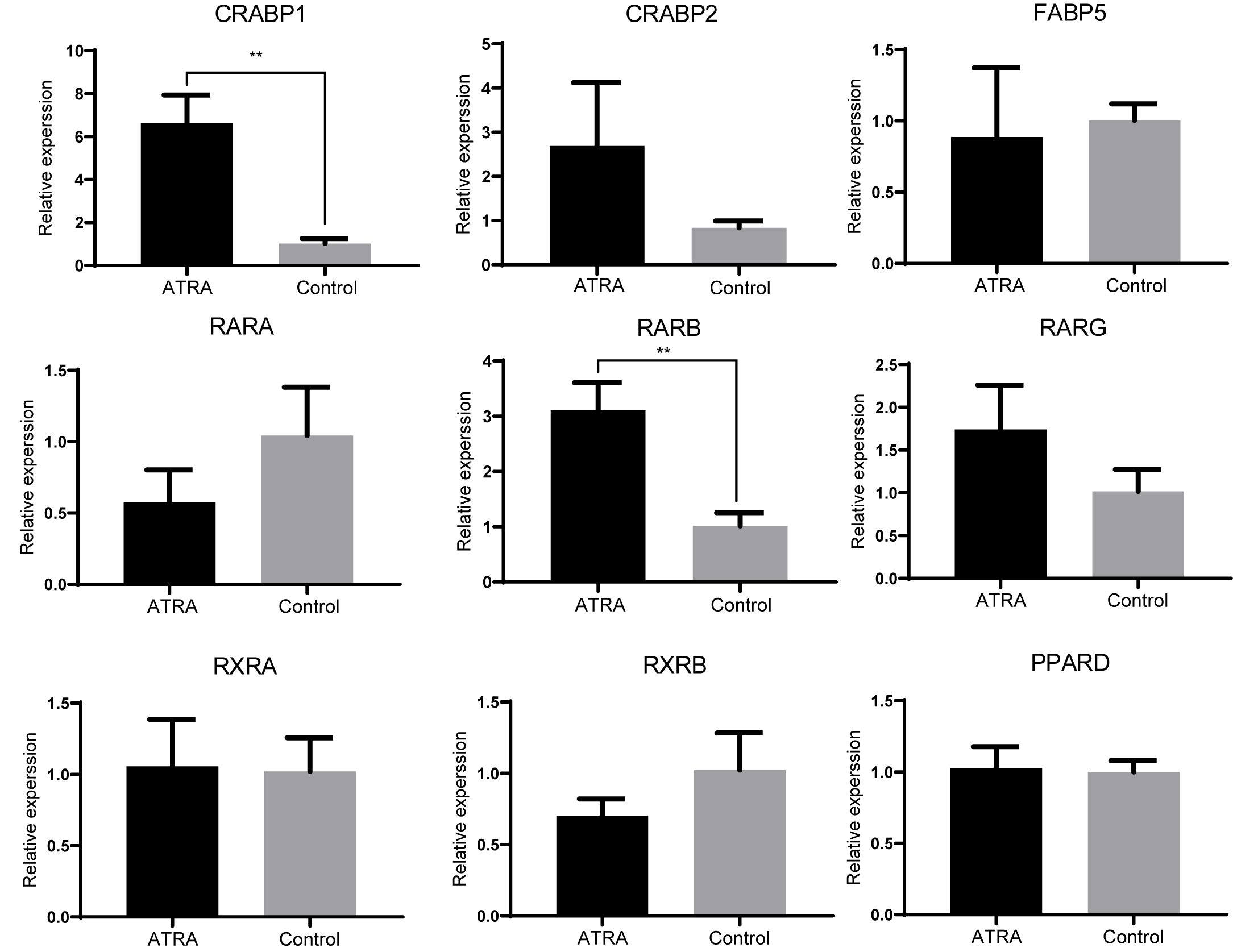

Supplement: Supplementary file 1 [file animals-10-01400-s001.zip › animals-842499-ffsup/Figure S1 Varied expression of ATRA signaling pathway-related genes in goat HMCs after ATRA treatment. .tif]

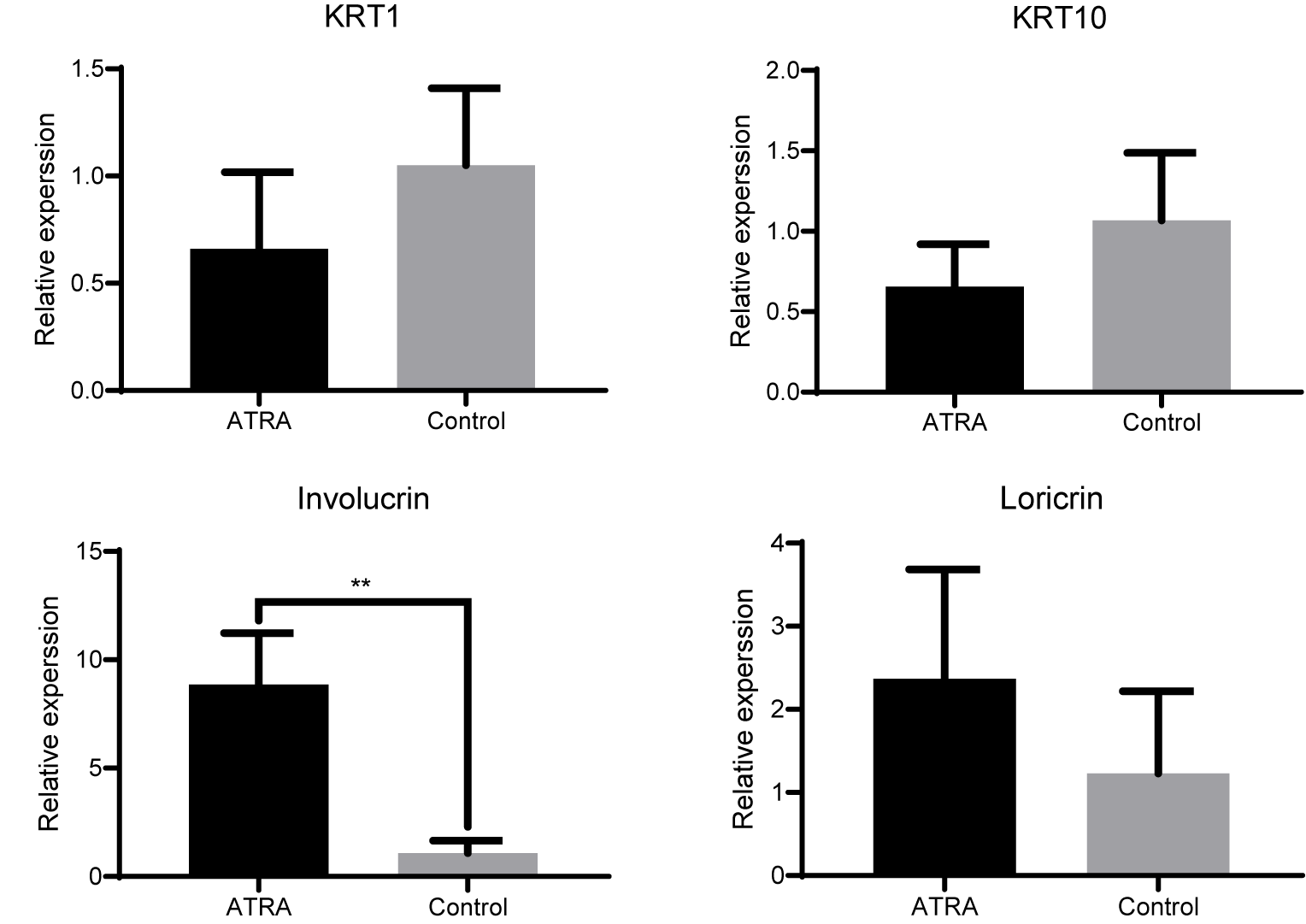

Supplement: Supplementary file 1 [file animals-10-01400-s001.zip › animals-842499-ffsup/Figure S2 Expression of marker genes for keratinocyte differentiation in goat HMCs after ATRA treatment..tif]
